# The intersecting effects of race, wealth, and education on AIDS incidence, mortality, and case-fatality rate: a Brazilian cohort study of 28.3 million individuals

**DOI:** 10.21203/rs.3.rs-4314004/v1

**Published:** 2024-05-10

**Authors:** Iracema Lua, Laio Magno, Andréa Silva, Priscila Pinto, João Luiz Bastos, Gabriela Jesus, Ronaldo Coelho, Maria Ichihara, Mauricio Barreto, Carlos Teles Santos, Corrina Moucheraud, Pamina Gorbach, James Macinko, Luis Souza, Inês Dourado, Davide Rasella

**Affiliations:** Institute of Collective Health, Federal University of Bahia (UFBA); Department of Health, State University of Feira de Santana (UEFS); Department of Life Sciences, State University of Bahia (UNEB). Institute of Collective Health, Federal University of Bahia (UFBA).; Institute of Collective Health, Federal University of Bahia; Center for Data and Knowledge Integration for Health (CIDACS), Gonçalo Moniz Institute, Oswaldo Cruz Foundation (FIOCRUZ), Salvado; Institute of Collective Health, Federal University of Bahia; Center for Data and Knowledge Integration for Health (CIDACS), Gonçalo Moniz Institute, Oswaldo Cruz Foundation (FIOCRUZ), Salvado; Faculty of Health Sciences, Simon Fraser University; Faculty of Medicine, Federal University of Bahia (UFBA); Department of Chronic Conditions, Diseases, and Sexually Transmitted Infections at the Brazilian Ministry of Health; The Center for Data and Knowledge Integration for Health (CIDACS), Oswaldo Cruz Foundation (FIOCRUZ); Center for Data and Knowledge Integration for Health (CIDACS), Gonçalo Moniz Institute, Oswaldo Cruz Foundation (FIOCRUZ); Institute of Collective Health, Federal University of Bahia, Salvador, Brazil; Center for Data and Knowledge Integration for Health (CIDACS), Fiocruz, Salvador, Brazil; Departments of Health Policy and Management and Community Health Sciences, UCLA Fielding School of Public Health; Department of Epidemiology, UCLA Fielding School of Public Health; Department of Health Policy and Management, University of California; Institute of Collective Health, Federal University of Bahia (UFBA).; Institute of Collective Health, Federal University of Bahia (UFBA); ISGlobal

## Abstract

The relationships between race, education, wealth, their intersections and AIDS morbidity/mortality were analyzed in retrospective cohort of 28.3 million individuals followed for 9 years (2007–2015). Together with several sensitivity analyses, a wide range of interactions on additive and multiplicative scales were estimated. Race, education, and wealth were each strongly associated with all of the AIDS-related outcomes, and the magnitude of the associations increased as intersections were included. A significantly higher risk of illness (aRR: 3.07, 95%CI:2.67–3.53) and death (aRR: 4.96, 95%CI:3.99–6.16) from AIDS was observed at the intersection of Black race, lower educational attainment, and less wealth. A higher case-fatality rate (aRR: 1.62, 95%CI:1.18–2.21) was also seen for the same intersectional group. Historically oppressed groups lying at the intersections of race, education, and wealth, had a considerably higher risk of illness and death from AIDS. AIDS-related interventions will require the implementation of comprehensive intersectoral policies that follow an intersectionality perspective.

## Introduction

Health inequities stem from historical processes of oppression and marginalization.^1^ In Latin American countries, slavery, patriarchy, and colonialism are the major social forces through which large population segments have been marginalized and oppressed over time.^2^ Groups that experience social exclusion–including Black and Brown populations, migrants, women, illiterate people, or those with lower educational attainment – exhibit lower levels of income, a limited capacity to transfer wealth to the next generation, and lower social standing,^3^ which can ultimately lead to worse health and well-being.^1–3^

An intersectionality perspective – which assumes that historical contexts, power relations, and structural forms of oppression shape individual and group experiences – has increasingly been used to understand complex patterns of health inequities.^1^ Applying an intersectional approach to health inequities highlights how the interaction between multiple social categories (i.e., the social markers of difference, such as race, gender, social class etc.) may reflect interconnected broad and overlapping of oppression/privilege systems (e.g., racism, sexism, heterosexism, classism, etc.).^1,4–6^ The language “social markers of difference” is a terminology from social sciences that intends to define and understand how experiences of inequality are produced and operate for the production of social differences and hierarchies.^7^

HIV/AIDS researchers have long studied the health effects of social markers on a range of outcomes, most notably race,^8,9^ education^8,10^ and poverty/wealth.^9,10^ However, scant research on the health effects of their intersections has been conducted to date.^11–14^ Some studies have examined the intersections between gender, race, sexual orientation,^11,12^ and transphobia^14^ with: risk factors to sexually transmitted infections (STIs) and HIV infection,^11,14^ HIV and STI prevention,^12^ and retention in healthcare,^13^ revealing complex patterns of health inequities in countries with high levels of racism and ethnic segregation.

This study used the largest cohort of vulnerable individuals in Low- and Middle-Income Countries (LMIC) to apply the intersectionality perspective on health inequities, examine the relationships between the most relevant social markers of difference - i.e., race, education, and wealth, their intersections, and AIDS-related outcomes in a large sample of the Brazilian population. We focused on AIDS incidence, mortality, and case-fatality rates, and assumed that inequities in these outcomes are largely determined by historical and social processes that unequally distribute power across groups in contextually-specific ways.

## RESULTS

Among the 28,3 million individuals in the study cohort, 7,113,769 (29.08%) belong to the intersection analysis subgroups, 5,267 of whom are people living with AIDS (PLWA). Black individuals, those with lower educational attainment and lower wealth were concentrated in the country’s poorest regions: North and Northeast. They also had the worst household conditions, and had a higher proportion of PLWA who had not initiated antiretroviral therapy (ART) (Supplementary Table S3 in appendix, pp. 11–15).

### AIDS incidence

AIDS incidence rate was 20.92 per 100,000 person-years (PY), being higher among Black people (aRR: 1.54, 95%CI: 1.45–1.63), and participants with lower educational attainment (aRR: 1.52, 95%CI: 1.44–1.59), or less wealth (aRR: 1.34, 95%CI: 1.27–1.42). The incidence was even higher (Supplementary Figure S3 in appendix, p. 28) for individuals lying at the intersections of the social markers of difference (i.e., Black and lower educational attainment and wealth) (aRR: 3.07, 95%CI: 2.67–3.53); positive additive (S_111_: 1.58) and multiplicative (MIM_111_: 1.04) interactions were observed (Table 1).

### AIDS mortality

AIDS-related mortality rate was 6.90 per 100,000 PY, and was higher among Black individuals (aRR: 1.73, 95%CI: 1.60–1.87), those with lower educational attainment (aRR: 2.08, 95%CI: 1.92–2.26) or less wealth (RRa: 1.56, 95%CI: 1.39–1.75). A greater strength of association was observed among participants lying at the intersection of these markers (Supplementary Figure S3 in appendix, p. 28), with a higher risk of death observed among those who are Black and have lower educational attainment and wealth (aRR: 4.96, 95%CI: 3.99–6.16); positive additive (RERI_111_: 1.29; AP_111_: 0.26; S_111_: 2.38) and multiplicative (MIM_111_: 1.35) interactions were observed (Table 2).

### AIDS case-fatality rate

The case-fatality rate was 6.67 per 100 PY, with a higher proportion of death among PLWA who were Black (aRR: 1.20, 95%CI: 1.06–1.36), with lower educational attainment (aRR: 1.39, 95%CI: 1.24–1.56), and less wealth (aRR: 1.02, 95%CI: 0.86–1.23). A higher case-fatality rate was estimated at the intersection of these markers, with a higher risk of death among those who are Black with lower educational attainment and wealth (aRR: 1.62; 95%CI: 1.18–2.21); positive additive (S_111_: 2.95) and multiplicative (MIM_111_: 1.39) interactions were observed (Table 3).

### Analyses stratified by gender

After stratification of the analyses by gender, Black women with lower educational attainment and wealth had higher AIDS incidence (39.09 per 100,000 PY) and mortality rates (15.53 per 100,000 PY). In addition, they had nearly five times higher risk of being an AIDS case (aRR: 4.83, 95%CI: 4.09–5.69), compared to White women with higher educational attainment and wealth. Black men with lower educational attainment and wealth had twice the risk (aRR: 2.11, 95%CI: 1.79–2.48), compared to White men with higher educational attainment and wealth. Positive additive interactions were identified in both analyses, with more expressive measures among women (S_111_: 1.74) than men (S_111_: 1.66) (Table 4).

Similar patterns are observed for AIDS-related mortality: Black women with lower educational attainment and wealth had seven times higher risk of dying from AIDS (aRR: 7.55, 95%CI: 5.71–9.98), compared to White women with higher educational attainment and wealth. Men had 3 times higher risk of an AIDS-related death (aRR: 3.52, 95%CI: 2.64–4.69), with positive additive interactions for both, but stronger among women (S_111_: 2.67) than men (S_111_: 2.36) (Table 4). For PLWA, the highest risk of an AIDS-related death was observed among men at the intersection of the three social markers of difference; they had 70% higher risk (aRR: 1.70, 95%CI: 1.11–2.61), compared to white men with higher educational attainment and wealth (S_111_: 1.08). This association was not statistically significant among women (Table 4).

### Sensitivity analyses

All sensitivity analyses demonstrated that the findings were consistent, even after fitting models with alternative structures and specifications. The analyses restricted to municipalities with adequate QVI showed no significant changes in the model estimates (Supplementary Tables S5 to S11 in appendix, pp. 29–48).

## DISCUSSION

To our knowledge, this is most extensive and comprehensive intersectionality study ever developed, thanks to the use of the largest cohort of vulnerable individuals available in LMIC and a highly diverse array of interaction measures. We were able to show that groups experiencing racial-, educational-, and poverty-based oppression had a significantly higher risk of illness and death from AIDS, when compared to their socially privileged peers. Each of these social markers of difference was strongly associated with the study outcomes but were more likely to explain the variability of AIDS-related conditions when analyzed according to an intersectionality perspective. Furthermore, an increase of these risks was observed when assessing gender differences, revealing that Black women with lower wealth and lower educational attainment suffer considerably more from AIDS-related outcomes.

The strength of our empirical approach to evaluate intersectionality is based on the use of a wide range of indicators, which allow us to show the existence of positive and high magnitude interactions between the three social markers of difference assessed. We were able to identify an excess risk ratio of dying from AIDS (RERI: 1.29) for individuals who are black, have lower educational attainment and wealth, which is considerably higher in case they are women (RERI: 3.10), providing evidence of a mechanistic interaction (according to sufficient causality or “epistatic interaction”).^24^

The intersections between these various social markers of difference go beyond merely adding or multiplying the effects of isolated factors, and allow a more comprehensive analysis of social determination.^26^ The strong effects on AIDS incidence in these groups show us two ways in which oppression and marginalization may operate: a) greater risk of infection by HIV, that is, the most vulnerable individuals have a worse understanding and ability to act on health information (including the importance of prevention, testing, and treatment), and are more exposed to sexual risk (monetary or nonmonetary exchange sex); and b) advance of the disease, since the health systems are not reaching these groups for early diagnosis and treatment. The increased risk of AIDS deaths reaffirms that these groups are also being left behind in the access and continuum of HIV/AIDS care within the healthcare system, which should be universal and equitable. Where is the equity, since the more privileged have more access and the more vulnerable certainly suffer discrimination for entry into these services?

The broader context of marginalized and oppressed groups may explain these results. Global studies indicate that Black people have worse HIV/AIDS-related outcomes.^4,18,27^ There is no evidence for the existence of genetic factors to account for this reality, reinforcing the hypothesis that racism is responsible for worse health outcomes^1,2^, including higher HIV rates,^28^ lower ART use,^9^ and poor adherence to ART.^8^ Moreover, racism is also a driver of social exclusion, and less access to education and income, which could provide a better quality of life,reinforcing the vicious cycle of oppression^29^ and structural violence that prevent individuals or groups from reaching their full potential.^1^ Our findings also showed that women suffer more from the effects of social processes.^5^ The intersection of racism and sexism, as well as other systems of oppression, shapes life experiences and opportunities.^1,30^

### Strengths and limitations

This study has some limitations. The first is the composition of the cohort under study, which represents the Brazilian population with the lowest socioeconomic status. However, this also represents a strength since it includes extremely poor and marginalized individuals who are usually underrepresented in health research. Another limitation faced by the authors was the lack of comparable studies in the literature and of an established protocol for intersection analyses in quantitative studies with large cohorts of individuals. Many approaches are used, but a unified and consolidated methodology for studying intersectionality is lacking. Its inclusion in quantitative research is still recent, although its great potential for public health is already recognized. The lack of quantitative guidelines has also brought limitations to the inclusion of a fourth intersection category. Efforts were made to adapt the existing formulas usually adopted in case of two intersections for the inclusion of a third intersection, however it was not possible to adapt formulas for a fourth intersection, and stratified analyses were chosen instead.

Despite these limitations, the main strength of the study is the use of an unprecedented, large dataset cohort which allowed the evaluation of synergistic effects in various intersectional positions, providing an opportunity to add statistical power and a higher level of resolution to existing maps of social inequalities within populations,enabling the inclusion of new SDH in intersectional analyses to advance knowledge about how social identities intersect to determine different ways of illness and death, extending the classical triad of race, class and gender that is usually assessed by intersectional studies.. The wide availability of socioeconomic data in the cohort further allowed us to advance the necessary adjustments and ensure the possibility of interpretations that approximate causal interactions.^24^ To this end, a variety of individual, family, and municipal characteristics were included in the models.

Moreover, the inclusion of variables at the municipal level enables the consideration of heterogeneities present among Brazilian municipalities, whether in socioeconomic aspects or health service structure. Thus, sensitivity models were tested, including multilevel models and analyses restricted to municipalities with adequate quality of vital information, and no correlations at the aggregate level (municipal, state or regional) or changes in the estimates of the restricted models were identified (Supplementary appendix).

To mitigate the adverse health impacts of intersecting factors, coordinated public interventions addressing broader social processes should take place. In Brazil, implementation of poverty-reduction and primary health care policies, like the *Bolsa Familia* Program and Family Health Strategy, have been shown to be have a synergistic effect on the improvement of child health.^31^ Moreover, transversal policies of “racial quota” in public universities and other policies to provide access to higher education (such as the Unified Selection System-*Sisu*, University for All Program-*Prouni*, Student Financing Fund-*FIES*), have been shown to be effective in reducing racial and social class disparities in educational outcomes.

Our application of an innovative intersectionality approach to an unprecedently large cohort was able to evaluate how social markers of difference intersect and shape AIDS-related outcomes in a highly unequal country such as Brazil. Social differences impair individuals’ health, and the intersection between these factors synergistically amplify health inequities. The interlocking systems of oppression and marginalization must be comprehensively addressed in order to effectively reduce social and health inequities. Acting only on the improvement of healthcare is not enough, and profound societal and political changes are urgently needed.

## METHODS

This retrospective cohort study uses a subsample of data from the 100 Million Brazilian Cohort,^15^ a consolidated cohort that used validated linkage algorithms^16^ to merge the Unified Registry of Brazilians (*CadÚnico*), a national tool used to determine eligibility for social protection programs, with the Brazilian National Surveillance System and the Brazilian Mortality Information System over a 9-year period. The Center for Data and Knowledge Integration for Health (CIDACS) developed this linkage (Supplementary Appendix).^15,16^ The study protocol^17^ was approved by the Research Ethics Committee of the Collective Health Institute at the Federal University of Bahia, number 41691315.0.0000.5030 (Assessment n°:3.783.920).

### Dataset and Study Population

We selected a sample of 28.3 million Brazilian people aged ≥13 years, and registered between 2007 and 2015 from the 100 Million Brazilian Cohort. Individuals diagnosed with AIDS, or those already deceased before the start of the cohort follow-up (January 1, 2007), were excluded (description of the selection process in the Supplementary Figure S1 in appendix, p. 4). For the intersectional analyses, three markers of social differences - race, education and wealth – were selected based on their relevance for HIV/AIDS outcomes,^8–10,18^ including their strong health effects observed in a previous study developed with the same cohort^18^, and other preliminary analyses (Supplementary Table S1 in appendix, pp. 5–6). Intersectional analyses were conducted using a binary classification of the social difference markers: race (Whites/Asian or Blacks), education (higher or lower educational attainment) and wealth (higher or lower wealth). Other race categories (i.e., Brown and Indigenous) and the intermediate level of wealth were not presented in the main study results but were included in the estimated models (Supplementary Table S2 in appendix, pp. 8–10).

### Variables and measures

Follow-up was calculated from the cohort entry date or AIDS diagnosis (case-fatality rates) until the time of censoring, which was determined by: a) AIDS diagnosis (incidence); b) AIDS-related death (mortality and case-fatality rates); c) death by other causes; or d) study end date (December 31, 2015) (Supplementary Chart S2 in appendix, p. 18). The criteria used to define AIDS cases were adapted CDC, the Rio de Janeiro/Caracas, and AIDS deaths. AIDS deaths were defined as the underlying cause of the ICD-10 codes B20 to B24 (Supplementary Charts S1 and S2 in appendix, pp. 16–18).

Associations between race, education, wealth, and AIDS-related outcomes were examined both individually and intersectionally. Thus, dummy variables were constructed with two- and three-way interaction terms, and categorized into: a reference group (Whites, higher educational attainment and wealth), groups with the presence of one social marker (isolated markers of social difference), and groups with an intersection of two or more social markers (combined markers of social difference) (Supplementary Figure S2 in appendix, p. 20). The following comparisons were thus analyzed: 1.

Intersection between wealth and education: individuals with higher wealth & higher education (reference group) were compared with individuals of: (a) higher wealth & lower education, (b) lower wealth & higher education (isolated markers of social difference), and (c) lower wealth & lower education (combined markers of social difference); 2. Intersection between race and education: White/Asian individuals with higher education (reference group) were compared with individuals of: (a) White/Asian race & lower education, (b) Black race & higher education (isolated markers of social difference), and (c) Black race & lower education (combined markers of social difference); 3. Intersection between race and wealth: White/Asian individuals with higher wealth (reference group) were compared with individuals of: (a) White/Asian race & lower wealth, (b) Black race & higher wealth (isolated markers of social difference), and (c) Black race & lower wealth (combined markers of social difference); 4. Intersection between race, education, and wealth: White/Asian individuals with higher wealth & higher education (reference group) were compared with individuals of: (a) White/Asian race & higher wealth & lower education, (b) White/Asian race & lower wealth & higher education, (c) Black race & higher wealth & higher education (isolated markers of social difference), (d) White/Asian race & lower wealth & lower education, (e) Black race & higher wealth & lower education, (f) Black race & lower wealth & higher education, and (g) Black race & lower wealth & lower education (combined markers of social difference) (Supplementary Figure S2 in appendix, p. 20).

Gender was taken as an effect-modifier of the association between race, wealth, education, and AIDS outcomes, and was used in the stratification analysis. The following individual-, household-, and municipal-level variables associated with both the independent variables and outcomes were included in the multivariate analyses: geographic factors (Brazilian geographic region and area of residence); time since last received conditional cash transfers (*Bolsa Família*), household conditions, age, year of admission into the cohort; and health infrastructure (primary health care coverage, number of specialized clinics, physicians, and hospital beds per 1,000 inhabitants), average AIDS outcome rate (in the follow-up period) under study, and socio-environmental conditions (Gini index, extreme poverty and unemployment rates) in municipality of residence (Figure 1).

### Statistical analysis

Descriptive statistics explored the distribution of the three-way intersection groups by the socioeconomic covariates. To estimate the crude (RR) and adjusted (aRR) rate ratios, multivariable Poisson regression models were used,^19,20^ using robust standard errors, clustered in the municipality of residence, and separately for each outcome. Poisson regression models that incorporate follow-up time as an offset variable to generate interpretable risk measures^21^ are common in cohort data analyses^22^ and have widely been used in similar observational studies using the 100 Million Brazilians Cohort.^18–20^

The intersections of markers of social differences were also evaluated through a wide range of interactions indicators on additive and multiplicative scales.^5,23^ This innovative approach allows to estimate the intersectionality in a more robust and comprehensive way, because it assigns a direction (positive, null, or negative) and a magnitude (assessing how much higher is the risk attributed to the combined characteristics) to the interaction under study, which is particularly useful for the implementation of more equitable public policies.^24^

We calculated several interaction measures: (a) Excess Risk (ER), that is, how much the risk of the presence of interaction exceeds the risk of none of the characteristics present;^23^ (b) Relative Excess Risk due to Interaction (RERI), that is, how much the excess risk due to the presence of interaction deviates from the null value (RERI>0 signals interaction);^23,25^ (c) Attributable Proportion (AP), which shows the proportion of cases due to the interaction of both exposures (AP>0 signals interaction);^23,25^ (d) Synergy Index (S), which reflects the direction of the interaction in relation to the null value (S=1 signals no interaction, S>1 synergy, and S<1 antagonism);^23^ (e) Multiplicative scale interaction measure for hazard ratios (MIM), which assesses the extent to which the effect of the interaction between exposures exceeds the product of the effects of the exposures seen separately (MIM>1 signals positive interaction, MIM<1 negative, MIM=1 null);^24^ and (f) Regression product terms, i.e. the RR estimate and the 95% confidence intervals (CI) obtained for the product term.^24^ Moreover, we evaluated at the same time three social markers, namely race, education, and wealth, which is innovative in intersectional research and even in HIV/AIDS research, and it has been possible because of our large data samples and our adaptations in the calculation of the indicators interaction (Supplementary Chart S3 in appendix, pp.22–23).

Adjusted Rate Ratios were calculated while controlling for all covariates (Figure 1). We implemented a number of strategies to assess the robustness of our findings (Supplementary Appendix, pp. 29–48): 1) Sensitivity analyses: alternative regression models were run (negative binomial regression; survival models, and multilevel models), to examine potential changes in the estimates based on model form specification; 2) Further adjustments for covariates: models were re-estimated with and without municipal-level variables; and 3) Data quality influence analyses: to determine the influence of municipal differences in surveillance data quality, we estimated models only for individuals living in municipalities with an adequate quality of vital information (QVI), according to validated criteria, and compared with the findings from all individuals under study. All analyses were performed using Stata^®^ 15.0.

### Role of the funding source

This study was funded by the National Institute of Allergy and Infectious Diseases - NAIDS/NIH, Grant Number: 1R01AI152938. The study funders played no role in the study design, data collection, data analysis, data interpretation, or writing of the paper. None of the authors were precluded from accessing study data, and they accepted the responsibility to submit the paper for publication.

## Data Availability

The protocol for creation of the 100 Million Brazilians Cohort, and the 100 Million Brazilians Cohort profile is available in the publications referenced in the article. Further material is available at: https://cidacs.bahia.fiocruz.br/en/platform/cohort-of-100-millionbrazilians. The linkage protocols are explained in the referenced publications, and the codes are available at: https://gitHub.com/gcgbarbosa/cidacs-rl. Individual-level data will not be available for sharing, due to confidentiality and ethical issues.
